# Estimated Glomerular Filtration Rate and Incident Prediabetes Risk in Normoglycemic Adults With Parental Type 2 Diabetes

**DOI:** 10.1210/jendso/bvaf160

**Published:** 2025-10-11

**Authors:** Blair Brawley, Louis Brown, Peace Asuzu, Samuel Dagogo-Jack

**Affiliations:** Division of Endocrinology, Diabetes and Metabolism, Department of Medicine, University of Tennessee Health Science Center, Memphis, TN 38163, USA; Division of Endocrinology, Diabetes and Metabolism, Department of Medicine, University of Tennessee Health Science Center, Memphis, TN 38163, USA; Division of Endocrinology, Diabetes and Metabolism, Department of Medicine, University of Tennessee Health Science Center, Memphis, TN 38163, USA; Division of Endocrinology, Diabetes and Metabolism, Department of Medicine, University of Tennessee Health Science Center, Memphis, TN 38163, USA; General Clinical Research Center, University of Tennessee Health Science Center, Memphis, TN 38163, USA

**Keywords:** kidney function, metabolic syndrome, euglycemic clamp, insulin secretion, insulin resistance, race/ethnicity

## Abstract

**Objective:**

We examined estimated glomerular filtration rate (eGFR) in relation to cardiometabolic and glucoregulatory factors and prediabetes risk in healthy subjects.

**Methods:**

Participants were normoglycemic Black and White offspring of parents with type 2 diabetes followed for 5 years in the Pathobiology of Prediabetes in a Biracial Cohort study. Baseline assessments included clinical examination, oral glucose tolerance test, blood chemistries, insulin sensitivity (Si-clamp), insulin secretion, and eGFR (derived from the CKD-EPI equation). We analyzed baseline eGFR in relation to metabolic syndrome (MetS), glucoregulatory function, and prediabetes risk using linear regression and Cox proportional hazards models.

**Results:**

The participants (n = 296; 73% female; 138 Black, 158 White) were aged 45.5 ± 10.1 years; body mass index (BMI) was 30.5 ± 7.6 kg/m^2^, and eGFR was 103 ± 18.7 mL/min. Baseline eGFR increased with cumulative MetS components (ANOVA *P* = .0002) and correlated significantly with waist circumference (r = 0.39, *P* < .0001), high-density lipoprotein cholesterol (r = −0.38, *P* < .0001), Si-clamp (r = −0.22; *P* = .003), and insulin secretion (r = 0.22; *P* = .0003). Higher baseline eGFR predicted lower risk of incident prediabetes: hazard ratio 0.986 (95% confidence interval 0.975-0.997, *P* = .012), adjusted for age, sex, ethnicity, BMI, waist circumference, glucose, insulin sensitivity, insulin secretion, and albuminuria.

**Conclusion:**

eGFR variations within the normal range signify cardiometabolic risk status, glucoregulatory function, and incident prediabetes risk among normoglycemic persons. Further studies are needed to determine the mechanisms linking kidney function and early dysglycemia.

Chronic kidney disease (CKD) and end-stage kidney disease are serious complications of diabetes mellitus. The development of CKD in people with diabetes amplifies the morbidity and mortality associated with either condition alone via mechanisms that involve glycemic and nonglycemic factors, including insulin resistance, inflammation, oxidative stress, endothelial and arterial dysfunction, hemodynamic stress, and hyperuricemia, among others [1-4]. Besides the well-known association between diabetes and CKD, there is evidence for an earlier proximal interaction between kidney function and regulation of glucose metabolism [5-8]. The healthy kidney plays an important role in glucose metabolism, with renal cortical cells contributing approximately 20% of total gluconeogenesis [5, 6]. The kidney also is a site of glucose uptake and utilization, especially by the medullary cells [5, 6]. The glucose-related metabolic processes in the kidney are under physiological regulation by insulin. Insulin inhibits renal gluconeogenesis, stimulates glucose uptake, and increases glucose reabsorption via sodium-glucose cotransporters (SGLT) [5-8]. Additionally, the kidney, as a major site of insulin clearance and catabolism, contributes to glucose homeostasis [9, 10].

Impaired kidney function often is associated with alterations in insulin dynamics, glucose tolerance, and glucose homeostasis, even in people without diabetes [5-8]. Conversely, altered metabolic signaling in the kidney can result in the activation of pathways promoting growth and hypertrophy, tissue remodeling, inflammation, fibrosis, and kidney dysfunction [11-13]. Moreover, hyperinsulinemia, a marker of insulin resistance, induces glomerular hyperfiltration and increased vascular permeability [11]. Metabolic syndrome, a manifestation of insulin resistance, is defined as the presence of at least 3 of 5 clinical criteria based on waist circumference, blood pressure, blood glucose, triglycerides, and high-density lipoprotein (HDL) cholesterol levels [14]. Metabolic syndrome and CKD share common clinical risk factors and related pathophysiological mechanisms [15-17].

Current classification of CKD is based on estimated glomerular filtration rate (eGFR) and albumin excretion: normal kidney function is indicated by eGFR values of ≥90 mL/min/1.73 m^2^ and urine albumin-to-creatinine ratios (uACR) of <30 mg/g [18]. Given the role of the kidney in glucose production and utilization and the effects of insulin/insulin resistance on renal structure and function, the goal of the present study was to examine the relationship between kidney function and cardiometabolic risk in people without diabetes or kidney disease. Specifically, we determined whether eGFR levels within the normal range are associated with insulin resistance and markers of metabolic syndrome in normoglycemic individuals. We also determined the association of baseline eGFR levels with the risk of incident prediabetes among initially normoglycemic participants in the Pathobiology of Prediabetes in a Biracial Cohort (POP-ABC) study followed up for 5 years [19].

## Materials and Methods

### Study Participants

We analyzed data from participants in the POP-ABC study, a prospective cohort study that enrolled non-Hispanic Black (African American) or non-Hispanic White (European American) adults, aged 18 to 65 years, with parental history of type 2 diabetes [19]. In addition to parental diabetes, eligible participants had normal fasting plasma glucose (FPG) [<100 mg/dL (5.6 mmol/L)]/normal 2-hour postprandial glucose (2hrPG) [<140 mg/dL (7.8 mmol/L)] during an oral glucose tolerance test (OGTT), as previously described [19]. Ineligible individuals were those with a diagnosis of diabetes or who were taking medications known to alter blood glucose, insulin secretion, insulin sensitivity, body weight, or albumin excretion [19]. The latter included any antihyperglycemic medication, glucocorticoids, thiazide diuretics, β-adrenergic blockers, prescription weight-loss drugs, angiotensin converting enzyme inhibitors, angiotensin II receptor blockers, and direct renin inhibitors. Enrolled participants were evaluated during quarterly follow-up visits for 5 years (mean 2.62 years). The POP-ABC study protocol was approved by the institutional review board of the University of Tennessee Health Science Center (approval #8399). All participants gave their written informed consent prior to the initiation of the study, which was conducted in accordance with the principles of the Declaration of Helsinki.

The primary outcome results from the POP-ABC study showed similar rates of incident prediabetes and glycemic progression among African American and European American offspring of parents with type 2 diabetes during 5 years of follow-up [20]. The present report is a post hoc analysis of the association of baseline eGFR levels with cardiometabolic risk factors in POP-ABC participants. Participants who had baseline serum creatinine and uACR values and evaluable data for pertinent cardiometabolic variables (waist circumference, blood pressure, glucose, lipids, insulin sensitivity, insulin secretion) were included in the present analysis. Participants were excluded if their baseline eGFR was less than 60 mL/min/or greater than 140 mL/min, to limit the cohort to individuals with healthy renal function without extreme hyperfiltration.

### Assessments

Participants were assessed at the General Clinical Research Center of the University of Tennessee Health Science Center. After fasting overnight, participants underwent baseline assessments, which included a medical history, a physical examination, and a 75-g standard OGTT [19, 20]. Weight, height, and waist circumference were measured using standard research protocols. The waist circumference was measured to the nearest 0.1 cm with a Gulick II tape measure (Country Technology, Inc., Gays Mills, WI, USA) at the midpoint between the lowest costal margin and the highest point of the iliac crest in the midaxillary line [19, 20]. The body mass index (BMI) was calculated as weight in kilograms divided by height in meters squared. An average of 2 blood pressure readings were recorded using an automated sphygmomanometer with the participant in the seated position and arm at chest level.

### Biochemical Measurements

Plasma glucose levels were measured using the YSI glucose analyzer (Yellow Spring Instruments Co., Yellow Springs, OH, USA). Hemoglobin A1c (HbA1c); fasting plasma lipid profiles, including HDL cholesterol and triglycerides; and serum creatinine levels were measured in a contract clinical laboratory utilizing standard methods.

### Insulin Sensitivity

The hyperinsulinemic euglycemic clamp procedure was used to quantify whole-body insulin sensitivity in overnight-fasted participants, as previously described [19, 21]. In brief, a primed, continuous IV infusion of regular human insulin (2 mU kg^−1^ min^−1^; 12 pmol kg^−1^ min^−1^) was given for 3 hours along with a variable infusion of dextrose (20%) to maintain euglycemia at ∼100 mg/dL (5.6 mmol/L). Glucose and insulin concentrations were assessed in bedside blood samples collected every 10 minutes. During the last 60 minutes of the insulin infusion (steady state), the rate of total insulin-stimulated glucose disposal (M) was calculated and corrected for the steady-state plasma insulin concentration to derive the insulin sensitivity index (Si-clamp) [21, 22].

### Insulin Secretion

Acute insulin secretory response to glucose (AIRg) was assessed using an IV glucose tolerance test, as previously described [19, 20, 23]. In brief, overnight-fasted participants were given an IV dextrose bolus (25 g), with arterialized blood sampling collected 30 minutes before and 2, 3, 4, 5, 7, and 10 minutes after dextrose administration. The AIRg was determined as the average incremental insulin level at 3, 4, and 5 minutes after the dextrose bolus [25, 26, 29]. The disposition index (insulin secretion corrected for ambient insulin sensitivity) was calculated as the product of Si-clamp and AIRg [19, 20, 23].

### Metabolic Syndrome Classification

Metabolic syndrome was defined using the National Cholesterol Education Program Adult Treatment Panel III criteria: HDL cholesterol < 50 mg/dL in women and <40 mg/dL in men; serum triglycerides ≥ 150 mg/dL; systolic blood pressure ≥ 130 mmHg or diastolic blood pressure ≥ 85 mmHg or drug treatment of hypertension; waist circumference ≥ 88 cm in women and ≥ 102 cm in men; and FPG ≥ 100 mg/dL [14]. As POP-ABC participants all had normal FPG or 2hrPG at enrollment, we utilized a modified metabolic syndrome definition based on the presence of at least 3 of the 4 nonglycemic criteria.

### Calculation of eGFR

The indexed eGFR was calculated automatically for each participant using the CKD epidemiology collaboration (CKD-EPI) creatinine equation (2021) at the National Kidney Foundation website interface [24, 25]. The indexed eGFR was then adjusted for body surface area (BSA) to obtain the nonindexed eGFR using the equation: [nonindexed glomerular filtration rate (GFR)] = [indexed GFR × BSA (m^2^)/1.73 m^2^]. The BSA was derived from the height (H) and weight (W) of participants using the DuBois and DuBois equation: [BSA(m^2^) = 0.007184 × W^0.425^ × H^0.725^], where H is measured in centimeters and W in kilograms. The nonindexed eGFR value (representing the actual estimate for each individual) was used for all analyses in the present report.

### Definition of Outcome

Study participants were seen in follow-up visits every 3 months at the General Clinical Research Center for 5 years (mean 2.62 years). The primary outcome was the occurrence of prediabetes, as indicated by impaired fasting glucose (IFG) (FPG 100-125 mg/dL) or impaired glucose tolerance (IGT) (2hrPG 140-199 mg/dL) based on American Diabetes Association criteria [19, 20]. All endpoints were confirmed using a standard 75-gram OGTT (usually performed within 6 weeks of initial endpoint occurrence) and adjudicated independently by the Institutional Data and Safety Officer (Murray Heimberg, MD, PhD).

### Statistical Analysis

Data are presented as means ± SD unless otherwise stated. Our study population showed skewed distributions for eGFR, uACR, triglycerides, AIRg, Si-clamp, and disposition index and normal distributions for the other variables. Continuous variables were analyzed using unpaired *t*-tests for variables that showed normal distribution and the Kolmogorov–Smirnov test for variables that were not normally distributed. Chi-square tests were used to analyze categorical variables, respectively. Linear regression models and Pearson's correlations were used to analyze the relationship between continuous variables, and Spearman's correlation was used to assess the monotonic directional relationship between ranked or ordinal variables. ANOVA with post hoc Fisher's least significant difference was used to analyze data from more than 2 comparison groups. The relationship between baseline eGFR and incident prediabetes was analyzed using Cox proportional hazards regression model, adjusted for baseline variables. We stratified participants by tertiles of baseline eGFR [tertile 1: 60-86.5 mL/min (n = 61), tertile 2: > 86.5-113 mL/min (n = 149), and tertile 3: > 113-140 mL/min (n = 86)] and used a Kaplan–Meier survival plot and log-rank test to analyze prediabetes probability across the tertiles. All analyses were conducted using Statview 5.0 statistical software (SAS Institute Inc., Cary, NC, USA), and *P* < .05 was deemed significant.

## Results

### Cohort Characteristics

Table 1 summarizes the baseline characteristics of study participants. The present study included 296 POP-ABC participants with evaluable baseline data for eGFR, uACR, and pertinent cardiometabolic variables (waist circumference, blood pressure, glucose, lipids, insulin sensitivity, and insulin secretion). The sample included 158 non-Hispanic Black or African American and 138 non-Hispanic White or European American participants, with a mean age of 45.5 ± 10.1 years and BMI of 30.5 ± 7.6 kg/m^2^. The cohort's mean baseline values for FPG, 2hrPG, HbA1c, blood pressure, triglycerides, HDL cholesterol, uACR, and eGFR were all within the normal reference ranges. The mean baseline values for blood pressure, BMI, waist circumference, 2hrPG, and HDL cholesterol did not differ significantly between African American and European American participants. However, age, FPG, triglycerides, and insulin sensitivity (Si-clamp) were lower, and HbA1c and insulin secretion (AIRg) were higher, in African American participants vs European American participants (Table 1). Of the 296 POP-ABC participants included in the present report, 31 were taking lipid-lowering medications at baseline: statins (n = 23), ezetimibe (n = 3), niacin (n = 2), ezetimibe + simvastatin fixed dose combination (n = 2), and fenofibrate (n = 1). The low number of participants taking lipid-lowering drugs is in keeping with a cohort selected for overall general good health. The 31 participants taking lipid-lowering medications at baseline were distributed as follows: subjects with metabolic syndrome (n = 9) vs those without metabolic syndrome (n = 22); progressors to prediabetes (n = 19) vs nonprogressors (n = 12); Black participants (n = 9) vs White participants (n = 22).

**Table 1.** Baseline characteristics of study participants by race/ethnicity

Characteristic	All	Black	White	*P-*value
Number (female/male)	296 (216/80)	158 (120/38)	138 (96/42)	.22
Age (years)	45.5 ± 10.1	43.7 ± 9.62	47.6 ± 10.2	.0009
Waist circumference (cm)	92.9 ± 14.3	93.5 ± 13.8	92.1 ± 14.8	.40
BMI (kg/m^2^)	30.5 ± 7.57	30.8 ± 7.95	30.2 ± 7.16	.50
Systolic BP (mmHg)	123 ± 16.3	125 ± 16.9	122 ± 15.5	.11
Diastolic BP (mmHg)	74.2 ± 9.12	75.1 ± 9.29	73.1 ± 8.84	.060
FPG (mg/dL)	91.9 ± 6.91	90.9 ± 7.24	93.0 ± 6.36	.0088
2hrPG (mg/dL)	122 ± 23.0	122 ± 24.3	123 ± 21.5	.73
HbA1c (%)	5.55 ± 0.45	5.65 ± 0.49	5.44 ± 0.39	<.0001
HDL cholesterol (mg/dL)	53.0 ± 13.4	53.5 ± 13.9	52.5 ± 12.9	.52
Triglycerides (mg/dL)	94.1 ± 55.6	76.9 ± 39.1	114 ± 64.6	<.0001
Si-clamp (µmol/kgFFM-min/pM)	0.14 ± 0.07	0.13 ± 0.07	0.14 ± 0.06	.044
AIRg (µU/mL)	83.4 ± 71.9	105 ± 88.3	59.7 ± 36.1	<.0001
Disposition index	4.88 ± 3.96	5.54 ± 4.76	4.26 ± 2.91	.031
eGFR (mL/min)	103 ± 18.7	103 ± 18.0	102 ± 19.6	.49
uACR (mg/g)	6.41 ± 4.90	6.26 ± 4.81	6.58 ± 5.01	.60

To convert the values for glucose to millimoles per liter, multiply by 0.0555. To convert the values for insulin to picomoles per liter, multiply by 7.175. To convert the values for triglycerides to millimoles per liter, multiply by 0.01129. To convert the values for HDL cholesterol to millimoles per liter, multiply by 0.02586.

Abbreviations: 2hrPG, 2-hour postload plasma glucose; AIRg, acute insulin response to IV glucose; BMI, body mass index; BP, blood pressure; eGFR, estimated glomerular filtration rate; FPG, fasting plasma glucose; HbA1c, hemoglobin A1c; HDL, high-density lipoprotein; Si-clamp, insulin sensitivity measured with hyperinsulinemic euglycemic clamp; uACR, urinary albumin-to-creatinine ratio.

### Distribution of Baseline eGFR Levels

**Figure 1.**
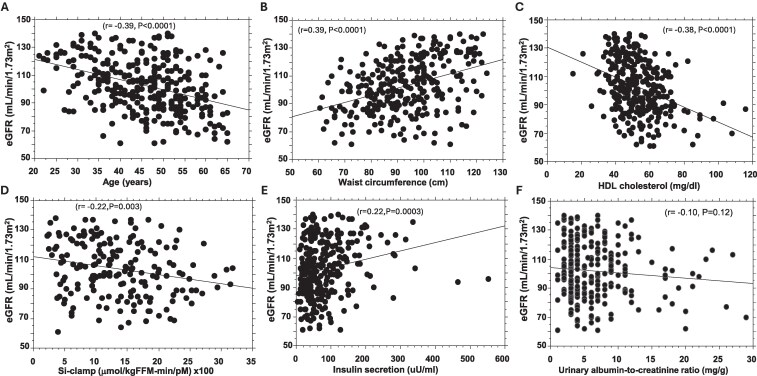
Linear regression of baseline estimated glomerular filtration rate vs age (A), waist circumference (B), high-density lipoprotein cholesterol (C), insulin sensitivity (Si-clamp, D), insulin secretion (E), and urinary albumin-to-creatinine ratio (F).

The mean baseline eGFR for the cohort was 103 ± 18.7 mL/min, with a median of 102 mL/min (interquartile range 29 mL/min). The mean baseline eGFR level was higher in men than women (110 ± 17.3 vs 100 ± 18.6 mL/min, *P* = <.0001) but similar in African American vs European American participants (103 ± 18.0 vs 102 ± 19.6 mL/min, *P* = .49). As expected, baseline eGFR levels were inversely correlated with age (r = −0.39, *P* = <.0001) (Fig. 1A). There was no significant association between baseline eGFR and uACR levels (r = −0.10, *P* = .12) (Fig. 1E).

### Baseline eGFR and Metabolic Syndrome

Table 2 compares demographic, clinical, and cardiometabolic variables in participants with metabolic syndrome (ie, those who harbored ≥3 components) vs participants without metabolic syndrome (ie, those with fewer than 3 components). The mean age and BMI were not significantly different between groups, but as expected, significant differences in metabolic syndrome components were observed based on the presence or absence of metabolic syndrome (Table 2). Although POP-ABC participants were enrolled with normoglycemia, participants with metabolic syndrome had higher baseline mean FPG and 2hrPG values and lower insulin sensitivity (Si-clamp) compared with those without metabolic syndrome (Table 2). Mean values for HbA1c and insulin secretion (AIRg) were not significantly different between the 2 groups (Table 2).

**Table 2.** Comparison of participants with metabolic syndrome vs participants who did not meet criteria for metabolic syndrome

Characteristic	All	Metabolic syndrome	No metabolic syndrome	*P*-value
Number (Black/White)	296 (158/138)	46 (20/26)	250 (138/112)	.143
Female/male	216/80	35/11	181/69	.60
Age (years)	45.5 ± 10.1	47.7 ± 9.98	45.1 ± 10.1	.11
Waist circumference (cm)	92.9 ± 14.3	104 ± 10.5	90.8 ± 13.9	<.0001
BMI (kg/m^2^)	30.5 ± 7.57	30.3 ± 8.14	30.6 ± 7.47	.81
Systolic BP (mmHg)	123 ± 16.3	137 ± 12.2	121 ± 15.7	<.0001
Diastolic BP (mmHg)	74.2 ± 9.12	79.5 ± 7.68	73.2 ± 9.05	<.0001
HDL (mg/dL)	53.0 ± 13.4	41.8 ± 7.74	55.1 ± 13.2	<.0001
Triglycerides (mg/dL)	94.1 ± 55.6	148 ± 68.2	84.3 ± 46.9	<.0001
FPG (mg/dL)	91.9 ± 6.91	93.6 ± 6.01	91.6 ± 7.02	.063
2hrPG (mg/dL)	122 ± 23.0	129 ± 21.5	121 ± 23.1	.02
HbA1c (%)	5.55 ± 0.45	5.57 ± 0.59	5.55 ± 0.43	.79
Si-clamp (µmol/kgFFM-min/pM)	0.13 ± 0.07	0.10 ± 0.06	0.14 ± 0.07	.0004
AIRg (µU/mL)	83.4 ± 71.9	100 ± 69.6	80.2 ± 72.1	.10
Disposition index	4.88 ± 3.96	5.08 ± 3.98	4.08 ± 3.80	.18
uACR	6.41 ± 4.90	6.44 ± 4.75	6.27 ± 5.65	.83
eGFR (mL/min)	103 ± 18.7	112 ± 18.2	101 ± 18.4	.0002

To convert the values for glucose to millimoles per liter, multiply by 0.0555. To convert the values for insulin to picomoles per liter, multiply by 7.175. To convert the values for triglycerides to millimoles per liter, multiply by 0.01129. To convert the values for HDL cholesterol to millimoles per liter, multiply by 0.02586.

Abbreviations: 2hrPG, 2-hour postload plasma glucose; AIRg, acute insulin response to IV glucose; BMI, body mass index; BP, blood pressure; eGFR, estimated glomerular filtration rate; FPG, fasting plasma glucose; HbA1c, hemoglobin A1c; HDL, high-density lipoprotein; Si-clamp, insulin sensitivity measured with hyperinsulinemic euglycemic clamp; uACR, urinary albumin-to-creatinine ratio.

**Figure 2.**
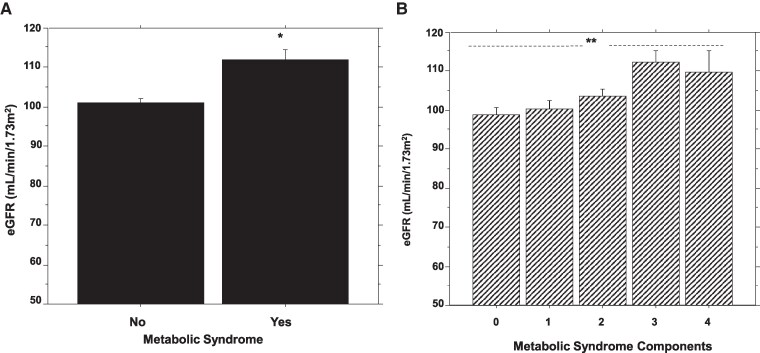
Comparison of baseline estimated glomerular filtration rate in participants with or without metabolic syndrome (A) and across participants with increasing burden of metabolic syndrome components (B). * *P* = .0002 vs no metabolic syndrome; *ANOVA *P* = .003.

The mean baseline eGFR level was significantly higher in participants with metabolic syndrome vs those who did not have metabolic syndrome (112 ± 18.2 vs 101 ± 18.4, *P* = .0002), adjusted for age, sex, and BMI (Table 2). Baseline eGFR tertiles and metabolic syndrome were significantly correlated (Spearman's ρ = 0.21, *P* = <.001). We observed significant univariate associations between eGFR values and 2 metabolic syndrome components (waist circumference and HDL cholesterol) (Fig. 1B and 1C). There was no significant association between eGFR and blood pressure (systolic r = 0.06, *P* = .32; diastolic r = 0.07, *P* = .23), triglycerides (r = 0.06, *P* = .30), or BMI (r = −0.01, *P* = .87). Baseline eGFR levels were lowest among participants who did not harbor a single component of metabolic syndrome (98.9 ± 17.0 mL/min) and increased progressively with increasing number of metabolic syndrome components (ANOVA *P* = .003) (Fig. 2).

### Association of eGFR Values With Insulin Sensitivity and Insulin Secretion

Values for insulin sensitivity determined using hyperinsulinemic euglycemic clamp (Si-clamp) at enrollment in the POP-ABC study were inversely correlated with baseline eGFR levels (r = 0.22; *P* = .003, Fig. 1D). In contrast, baseline values for insulin secretion (AIRg) were significantly positively correlated with baseline eGFR levels (r = 0.22; *P* = .0003) (Fig. 1E). There was no significant association between baseline eGFR and FPG (r = 0.07; *P* = .27), 2hrPG (r = 0.07; *P* = .24), or HbA1c (r = 0.04; *P* = .45) levels.

### Baseline eGFR and Progression to Prediabetes

During 5 years of follow-up (mean 2.62 years) in the POP-ABC study, 97 participants (32.8%) developed prediabetes (progressors) and 199 (67.2%) participants maintained normoglycemia (nonprogressors). Like the primary results from the entire POP-ABC study population, the present post hoc sample showed that progressors to prediabetes were older and had higher values for waist circumference, FPG, and 2hPG compared with nonprogressors (Table 3) [23]. Additionally, the progressors had lower baseline values for insulin sensitivity and disposition index vs nonprogressors (Table 3). We stratified participants into tertiles of baseline eGFR as follows: tertile 1: 60 to 86.5 mL/min (n = 61); tertile 2: >86.5 to 113 mL/min (n = 149); and tertile 3: >113 to 140 mL/min (n = 86). Table 4 summarizes baseline demographic, glycemic, and glucoregulatory variables across tertiles of baseline eGFR. There were no significant differences in BMI, fasting or postload plasma glucose, or HbA1c levels among participants in the different baseline eGFR tertiles. However, participants in the highest tertile of baseline eGFR had lower values for mean age and insulin sensitivity and higher values for waist circumference, insulin secretion, and disposition index compared with participant in lower tertiles.

**Table 3.** Baseline characteristics of participants with incident prediabetes (progressors) vs those who maintained normoglycemia (nonprogressors) during 5-year follow-up

Characteristic	All	Progressors	Nonprogressors	*P*-value
Number (Black/White)	296 (157/139)	97 (47/50)	199 (110/89)	.27
Age (years)	45.5 ± 10.1	48.1 ± 8.78	44.28 ± 10.5	.0024
BMI (kg/m^2^)	30.5 ± 7.57	30.2 ± 7.56	30.7 ± 7.57	.62
Waist circumference (cm)	92.9 ± 14.3	98.5 ± 12.3	90.1 ± 14.4	<.0001
FPG (mg/dL)	91.9 ± 6.91	94.1 ± 7.38	90.8 ± 6.44	.0001
2hrPG (mg/dL)	122 ± 23.0	129 ± 24.1	119 ± 21.9	.0008
Si-clamp (µmol/kgFFM-min/pM)	0.135 ± 0.07	0.115 ± 0.07	0.149 ± 0.06	.0006
AIRg (µU/mL)	83.4 ± 71.9	81.3 ± 76.1	84.3 ± 70.0	.75
Disposition index	4.88 ± 3.96	3.94 ± 2.83	5.58 ± 4.50	.006

To convert the values for glucose to millimoles per liter, multiply by 0.0555. To convert the values for insulin to picomoles per liter, multiply by 7.175. To convert the values for triglycerides to millimoles per liter, multiply by 0.01129.

Abbreviations: 2hrPG, 2-hour postload plasma glucose; AIRg, acute insulin response to IV glucose; BMI, body mass index; FPG, fasting plasma glucose; Si-clamp, insulin sensitivity measured with hyperinsulinemic euglycemic clamp.

**Table 4.** Baseline glycemic and glucoregulatory measures in participants stratified by baseline eGFR tertiles

Measure	Tertile 1	Tertile 2	Tertile 3	*P*-value
Age (years)	50.9 ± 9.46	46.2 ± 8.58	40.5 ± 10.7	<.0001
eGFR (mL/min)	77.3 ± 7.43	100 ± 8.36	126 ± 7.81	<.0001
Number (AA/EA)	61 (27/34)	150 (89/61)	85 (41/44)	.079
BMI (kg/m^2^)	30.8 ± 7.28	30.4 ± 6.71	30.5 ± 9.19	.942
Waist circumference	87.5 ± 15.0	90.6 ± 12.8	101 ± 12.9	<.0001
FPG (mg/dL)	91.9 ± 6.91	91.9 ± 7.79	91.2 ± 7.16	.764
2hrPG (mg/dL)	120 ± 20.3	122 ± 24.0	123 ± 23.2	.737
HbA1c (%)	5.55 ± 0.45	5.48 ± 0.38	5.58 ± 0.49	.198
Si-clamp (µmol/kgFFM-min/pM)	0.144 ± 0.06	0.142 ± 0.07	0.119 ± 0.06	.02
AIRg (µU/mL)	62.6 ± 45.9	82.1 ± 79.3	99.6 ± 71.0	.0009
Disposition index	4.03 ± 2.02	4.77 ± 3.68	5.56 ± 4.02	.032

To convert the values for glucose to millimoles per liter, multiply by 0.0555. To convert the values for insulin to picomoles per liter, multiply by 6.0. To convert the values for triglycerides to millimoles per liter, multiply by 0.01129.

Abbreviations: 2hrPG, 2-hour postload plasma glucose; AA, African American; AIRg, acute insulin response to IV glucose; BMI, body mass index; EA, European American; eGFR, estimated glomerular filtration rate; FPG, fasting plasma glucose; HbA1c, hemoglobin A1c; Si-clamp, insulin sensitivity measured with hyperinsulinemic euglycemic clamp.

**Figure 3.**
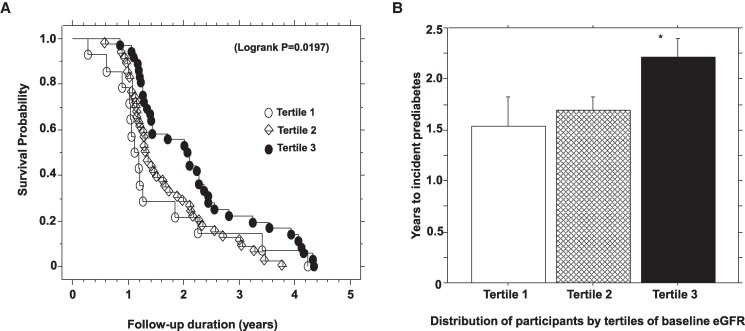
Kaplan–Meier plot of prediabetes survival probability (A) and mean time to prediabetes occurrence (B) by tertiles of baseline estimated glomerular filtration rate during 5-year follow-up in the POP-ABC study. *P = .02 vs tertiles 1 and 2.

In a Cox proportional hazards regression model, higher baseline eGFR was associated with lower risk of incident prediabetes: hazard ratio (HR) 0.986 [95% confidence interval (CI) 0.975-0.997, *P* = .012], adjusted for age at enrollment, sex, race/ethnicity, and baseline values for BMI, waist circumference, FPG, 2hPG, insulin sensitivity, insulin secretion, and uACR (Table 5). Kaplan–Meier analysis showed higher prediabetes survival probability associated with the highest tertile of baseline eGFR compared with lower tertiles during 5 years of follow-up in the POP-ABC study (log-rank *P* = .019) (Fig. 3). The mean time of progression from normoglycemia to incident prediabetes was 1.53 ± 1.09 years, 1.70 ± 0.81 years, and 2.22 ± 1.08 years among participants in baseline eGFR tertiles 1, 2, and 3, respectively (*P* = .022) (Fig. 3). Compared with participants in the lowest tertile of baseline eGFR, those in the highest tertile had 50% and 80% relative reduction in prediabetes risk in unadjusted and fully adjusted models, respectively (Table 5).

**Table 5.** Hazard ratios for incident prediabetes comparing participants in the lowest baseline eGFR tertile vs those in higher titles

	Referent: tertile1	Hazard ratio	95% CI	*P-*value
Unadjusted	Tertile 2	0.904	0.489-1.669	.75
	Tertile 3	0.510	0.272-0.954	.035
Model 1	Tertile 2	0.695	0.355-1.361	.29
	Tertile 3	0.392	0.193-0.795	.0094
Model 2	Tertile 2	0.603	0.299-1.213	.16
	Tertile 3	0.227	0.104-0.496	.0002
Model 3	Tertile 2	0.469	0.230-0.954	.037
	Tertile 3	0.189	0.087-0.410	<.0001
Model 4	Tertile 2	0.619	0.245-1.563	.31
	Tertile 3	0.209	0.076-0.572	.0023

Model 1: adjusted for age, race, sex.

Model 2: adjusted for age, race, sex, body mass index, waist circumference.

Model 3: adjusted for age, race, sex, body mass index, waist circumference, fasting plasma glucose, 2-hour postload plasma glucose.

Model 4: adjusted for age, race, sex, body mass index, waist circumference, fasting plasma glucose, 2-hour postload plasma glucose, insulin sensitivity, insulin secretion, urinary albumin-to-creatinine ratio.

Abbreviations: CI, confidence interval; eGFR, estimated glomerular filtration rate.

## Discussion

In our POP-ABC study population comprising normoglycemic individuals with overweight/obesity (mean BMI 30.5 kg/m^2^), higher baseline eGFR and presence of metabolic syndrome components were significantly correlated. A similar association between eGFR and cardiometabolic risk factors has been reported in other studies [26, 27]. Congruently, we found that eGFR levels correlated inversely with insulin sensitivity, with higher eGFR denoting greater insulin resistance, a key feature of metabolic syndrome. The participants with metabolic syndrome had significantly lower insulin sensitivity and numerically higher insulin secretion compared with participants who did not meet the criteria for metabolic syndrome. The relative hyperinsulinemia could be a mechanism for higher glomerular filtration, thus explaining our findings [28-30]. Insulin's actions in the kidney include stimulation of proliferation of renal cells and increased expression of growth factors and angiotensin II type 1 receptor in mesangial cells [31-34]. The latter would augment the vasoconstrictive effects of angiotensin II and endothelin-1 in the efferent arteriole, leading to hyperfiltration [33, 34]. A substantial body of literature links alterations in intrarenal insulin signaling to kidney dysfunction in animal models and people with or without diabetes [35-37]. Mouse models with targeted deletion of the podocyte insulin receptor show a phenotype characterized by loss of podocytes, attenuation of the foot processes, increased albuminuria, and glomerulosclerosis [37].

As already noted, we found an inverse association between eGFR and insulin sensitivity, consistent with previous reports in adults and children [26, 38, 39]. Naderpoor et al examined the relationship between eGFR and insulin sensitivity measured with the hyperinsulinemic euglycemic clamp technique in 104 healthy adults with overweight/obesity [38]. After adjustment for sex, BMI, and other potential variables, the investigators observed an inverse association between eGFR and insulin sensitivity [38]. One discordant study reported a positive association between GFR (calculated from serum cystatin C levels) and insulin sensitivity measured with hyperinsulinemic euglycemic [40]. Notably, that report emanated from an older (mean age 71 years), leaner (mean BMI 26.2 kg/m^2^), all-male Swedish cohort that included individuals with diabetes, IFG, or IGT and a substantial burden of comorbidities [40]. In contrast, our present findings were derived from a more diverse (men, women, African American, European American), younger (mean age 45.5 years), normoglycemic population with higher BMI (mean 30.5 kg/m^2^). Notably, baseline GFR values were lower among participants in the Swedish study vs our POP-ABC study (61.5 vs 103 mL/min) [40]. The other previous studies that reported an inverse association between eGFR and insulin sensitivity, like we found, also were conducted in younger, healthier, and more diverse populations [26, 38, 39]. As insulin sensitivity and kidney function both tend to decline with increasing age (and can be altered by numerous factors), the conflicting findings regarding the direction of association between insulin sensitivity and GFR likely reflect differences in the populations studied [26, 38-40].

Besides demonstrating the cross-sectional relationship between eGFR and metabolic syndrome/insulin resistance, the present study included a prospective component aimed at determining whether baseline eGFR was associated with the risk of progression from normoglycemia to prediabetes during 5 years of follow-up. Using a Cox proportional hazards model, we found that higher baseline eGFR predicted lower risk of progression to prediabetes [HR 0.986 (95% CI 0.975-0.997)]. In other words, participants enrolled with lower (albeit within the normal range) eGFR values were more likely to develop prediabetes during follow-up compared with those who had higher values. These finding suggest that variations in eGFR within the normal range among normoglycemic individuals, such as were enrolled in our POP-ABC study, conveyed prognostic information regarding the risk of incident prediabetes. As we found no significant association between baseline eGFR and glycemic markers (FPG, 2hrPG, HbA1c), any link between lower baseline eGFR and higher risk of prediabetes must be operating via indirect mechanisms. Notably, insulin secretion and the disposition index (insulin secretion corrected for ambient insulin sensitivity) were significantly lower in participants with lower baseline eGFR (tertiles 1 and 2) compared with those in the highest tertile of baseline eGFR. In previous reports from the POP-ABC study, higher disposition index at baseline was a stronger predictor of nonprogression from normoglycemia to prediabetes compared with insulin sensitivity or insulin secretion alone [20, 23]. Thus, robust insulin secretion could be 1 of the mechanisms that explain the protection against incident prediabetes that was observed among participants with higher baseline eGFR in the present study.

Our findings are concordant with the report by Wang et al, based on a large study population (n = 173 301 adults), which also found a significant association between higher baseline eGFR and lower risk of incident prediabetes during a mean follow-up period of 3 years [HR 0.993 (95% CI 0.992-0.995)] [41]. In another study, higher baseline eGFR significantly predicted reversion from prediabetes to normoglycemia among individuals enrolled with IFG during a follow-up period of 3 years [42]. However, the association between eGFR and prediabetes appears to be complex. In their study, Wang et al observed a U-shaped curve relationship between eGFR and prediabetes risk, with an eGFR inflection point of ∼130 mL/min/1.73 m^2^. The prediabetes HR for individuals with eGFR values below the inflection point was 0.993 (95% CI 0.991-0.994), and that for those above the inflection point was 1.023 (95% CI 1.010-1.037) [41].

Together, the present findings and previous reports associating lower eGFR with higher risk of dysglycemia implicate renal mechanisms in the pathogenesis of early glucose dysregulation. The kidney is a site of production, utilization, filtration, and reabsorption of glucose, as well as extraction and degradation of insulin [5-10]. In the kidney, the key enzymes for gluconeogenesis (pyruvate carboxylase, phosphoenolpyruvate carboxykinase, fructose-1,6-biphosphatase, glucose 6-phosphatase) are localized mainly in the cortical cells, and glycolytic enzymes (hexokinase, phosphofructokinase, pyruvate kinase) are located in the medulla [43]. The human liver and renal cortex contribute approximately equally to gluconeogenesis under fasting conditions. Postprandially, overall endogenous glucose production is markedly suppressed, but renal gluconeogenesis increases by approximately 60% [5, 6, 44]. Glucose consumption by the kidneys accounts for approximately 10% of whole-body glucose utilization during fasting and increases further postprandially [5, 6, 44]. Furthermore, the kidneys filter approximately 180 g of glucose daily and reabsorb nearly all of the filtered glucose via SGLT2 under normal conditions. Insulin promotes renal glucose uptake, suppresses renal gluconeogenesis, and regulates the expression of renal glycolytic enzymes and SGLT2 [5-8, 45, 46].

Through these processes, the kidneys contribute substantially to glucose homeostasis [5-14]. Dysregulated or maladaptive renal glucose handling in people with type 2 diabetes is evidenced by increased gluconeogenesis and increased glucose reabsorption via SGLT2 [5-10, 47]. Insulin resistance in the kidney is associated with glomerular hyperfiltration, increased gluconeogenesis, and alterations in renal glucose metabolism that predispose to the development of dysglycemia [11-13, 48, 49]. Indeed, the effects of metabolic dysfunction and insulin resistance on kidney function and the impact of a maladaptive renal environment on glucose homeostasis are discernible at the stage of prediabetes [48-50]. Our findings suggest that hemodynamic and metabolic mechanisms orchestrated by the kidney (likely mediated by insulin dynamics) might be involved in the pathogenesis of early glucose dysregulation leading to prediabetes.

We count the prospective design, enrollment of a diverse cohort, lengthy follow-up duration, and independent adjudication of prediabetes outcomes among the strengths of the present study. Additionally, we employed rigorous methodology for measuring insulin sensitivity. One limitation is the use of an indirect measure of GFR based on the CKD-EPI creatinine equation. Although widely used for estimating GFR in the clinical and research settings, and more accurate than other equations like the Modification of Diet in Renal Disease study equation, the CKD-EPI equation does have limitations. These include estimation errors in individuals with very large or very small body mass, decreased accuracy at the lower end of the eGFR range, and potential risk of overestimating or underestimating GFR depending on ethnic background [51]. Nonetheless, the CKD-EPI equation is recommended by experts and widely used in practice and research [24, 25]. Another limitation is that, by design, our POP-ABC study enrolled people with parental history of type 2 diabetes, which may limit the generalizability of our findings. Moreover, our findings were based on baseline assessments of eGFR, insulin sensitivity, and the other variables analyzed in the present study. Thus, the influence of time-dependent changes in those measures on incident prediabetes could not be determined.

Despite these limitations, the present study presents novel insights into the link between renal mechanisms and glucose homeostasis in otherwise healthy, normoglycemic, normotensive, and normoalbuminuric individuals. We had reported previously that POP-ABC participants who maintained normoglycemia during 5 years of follow-up (nonprogressors), in comparison with progressors to prediabetes, were characterized by younger age; lower BMI, waist circumference, and blood pressure; lower bone mineral density; higher levels of physical activity (adjusted for food habits); higher insulin sensitivity and secretion; higher adiponectin levels; and lower uACR levels at baseline [20, 23, 52-55]. The present report adds higher baseline eGFR as a significant predictor of nonprogression to prediabetes among African American and European American offspring of parents with type 2 diabetes.

## Data Availability

Some or all datasets generated and/or analyzed during the current study are not publicly available but are available from the corresponding author on reasonable request.

## References

[1] Morales J, Dagogo-Jack S, Fonseca V, Neumiller JJ, Rosas SE. Perspectives on chronic kidney disease with type 2 diabetes and risk management: practical viewpoints and a paradigm shift using a pillar approach. Clin Diabetes. 2023;41(4):553‐566. 37849516 10.2337/cd22-0110 PMC10577512

[2] Koye DN, Shaw JE, Reid CM, Atkins RC, Reutens AT, Magliano DJ. Incidence of chronic kidney disease among people with diabetes: a systematic review of observational studies. Diabet Med. 2017;34(7):887‐901. 28164387 10.1111/dme.13324

[3] Dagogo-Jack S . Screening, monitoring, prevention, and treatment strategies for chronic kidney disease in patients with type 2 diabetes. In: Weir Matthew R, ed. Chronic Kidney Disease and Type 2 Diabetes. American Diabetes Association; 2021:23‐27. 34279878

[4] Gupta S, Dominguez M, Golestaneh L. Diabetic kidney disease: an update. Med Clin North Am. 2023;107(4):689‐705. 37258007 10.1016/j.mcna.2023.03.004

[5] Gerich JE . Role of the kidney in normal glucose homeostasis and in the hyperglycaemia of diabetes mellitus: therapeutic implications. Diabet Med. 2010;27(2):136‐142. 20546255 10.1111/j.1464-5491.2009.02894.x PMC4232006

[6] Stumvoll M, Meyer C, Mitrakou A, Nadkarni V, Gerich JE. Renal glucose production and utilization: new aspects in humans. Diabetologia. 1997;40(7):749‐757. 9243094 10.1007/s001250050745

[7] Sharma R, Tiwari S. Renal gluconeogenesis in insulin resistance: a culprit for hyperglycemia in diabetes. World J Diabetes. 2021;12(5):556‐568. 33995844 10.4239/wjd.v12.i5.556 PMC8107972

[8] Legouis D, Faivre A, Cippà PE, de Seigneux S. Renal gluconeogenesis: an underestimated role of the kidney in systemic glucose metabolism. Nephrol Dial Transplant. 2022;37(8):1417‐1425. 33247734 10.1093/ndt/gfaa302

[9] Rubenstein AH, Mako ME, Horwitz DL. Insulin and the kidney. Nephron. 1975;15(3-5):306‐326. 1101090 10.1159/000180518

[10] Fawcett J, Rabkin R. Degradation of insulin by isolated rat renal cortical endosomes. Endocrinology. 1993;133(4):1539‐1547. 8404592 10.1210/endo.133.4.8404592

[11] Artunc F, Schleicher E, Weigert C, Fritsche A, Stefan N, Häring HU. The impact of insulin resistance on the kidney and vasculature. Nat Rev Nephrol. 2016;12(12):721‐737. 27748389 10.1038/nrneph.2016.145

[12] Whaley-Connell A, Sowers JR. Insulin resistance in kidney disease: is there a distinct role separate from that of diabetes or obesity? Cardiorenal Med. 2018;8(1):41‐49. 10.1159/000479801 PMC5757598 29344025

[13] De Cosmo S, Menzaghi C, Prudente S, Trischitta V. Role of insulin resistance in kidney dysfunction: insights into the mechanism and epidemiological evidence. Nephrol Dial Transplant. 2013;28(1):29‐36. 23048172 10.1093/ndt/gfs290

[14] Expert Panel on Detection, Evaluation, and Treatment of High Blood Cholesterol in Adults . Executive summary of the third report of the National Cholesterol Education Program (NCEP) expert panel on detection, evaluation, and treatment of high blood cholesterol in adults (Adult Treatment Panel III). JAMA. 2001;285(19):2486‐2497. 11368702 10.1001/jama.285.19.2486

[15] Lin L, Tan W, Pan X, Tian E, Wu Z, Yang J. Metabolic syndrome-related kidney injury: a review and update. Front Endocrinol (Lausanne). 2022;13:904001. 35813613 10.3389/fendo.2022.904001 PMC9261267

[16] Fanaei SM, Mehran L, Amouzegar A, Masoumi S, Amouzegar A, Azizi F. The impact of metabolic syndrome on chronic kidney disease development. Insights from a big prospective study. Eur J Clin Invest. 2023;53(4):e13945. 36576367 10.1111/eci.13945

[17] Ciardullo S, Ballabeni C, Trevisan R, Perseghin G. Metabolic syndrome, and not obesity, is associated with chronic kidney disease. Am J Nephrol. 2021;52(8):666‐672. 34569517 10.1159/000518111

[18] Kidney Disease: Improving Global Outcomes (KDIGO) CKD Work Group . KDIGO 2024 Clinical practice guideline for the evaluation, and management of chronic kidney disease. Kidney Int. 2024;105(4):S117‐S314. 38490803 10.1016/j.kint.2023.10.018

[19] Dagogo-Jack S, Edeoga C, Ebenibo S, Chapp-Jumbo E; Pathobiology of Prediabetes in a Biracial Cohort (POP-ABC) Research Group. Pathobiology of Prediabetes in a Biracial Cohort (POP-ABC) study: baseline characteristics of enrolled subjects. J Clin Endocrinol Metab. 2013;98(1):120‐128. 23118422 10.1210/jc.2012-2902 PMC3537095

[20] Dagogo-Jack S, Edeoga C, Ebenibo S, Nyenwe E, Wan J; Pathobiology of Prediabetes in a Biracial Cohort (POP-ABC) Research Group. Lack of racial disparity in incident prediabetes and glycemic progression among black and white offspring of parents with type 2 diabetes: the pathobiology of prediabetes in a biracial cohort (POP-ABC) study. J Clin Endocrinol Metab. 2014;99(6):E1078‐E1087. 24628558 10.1210/jc.2014-1077 PMC5393483

[21] James D, Umekwe N, Edeoga C, Nyenwe E, Dagogo-Jack S. Multi-year reproducibility of hyperinsulinemic euglycemic clamp-derived insulin sensitivity in free-living adults: association with incident prediabetes in the POP-ABC study. Metabolism. 2020;109:154263. 32445642 10.1016/j.metabol.2020.154263 PMC7387175

[22] DeFronzo RA, Tobin JD, Andres R. Glucose clamp technique: a method for quantifying insulin secretion and resistance. Am J Physiol. 1979;237(3):E214‐E223. 382871 10.1152/ajpendo.1979.237.3.E214

[23] Edeoga C, Asuzu P, Wan J, Dagogo-Jack S. Insulin secretion, sensitivity, and clearance in normoglycemic Black and White adults with parental type 2 diabetes: association with incident dysglycemia. BMJ Open Diabetes Res Care. 2024;12(6):e004545. 10.1136/bmjdrc-2024-004545 PMC11683886 39719314

[24] National Kidney Foundation . CKD-EPI Creatinine Equation (2021). Accessed June 18, 2025. https://www.kidney.org/ckd-epi-creatinine-equation-2021

[25] National Kidney Foundation . eGFR Calculator. Accessed June 18, 2025. https://www.kidney.org/professionals/gfr_calculator

[26] Xargay-Torrent S, Puerto-Carranza E, Marcelo I, et al Estimated glomerular filtration rate and cardiometabolic risk factors in a longitudinal cohort of children. Sci Rep. 2021;11(1):11702. 34083639 10.1038/s41598-021-91162-x PMC8175594

[27] Liu G, Tao L, Zhu Q, Jiao X, Yan L, Shao F. Association between the metabolic score for insulin resistance (METS-IR) and estimated glomerular filtration rate (eGFR) among health check-up population in Japan: a retrospective cross-sectional study. Front Endocrinol (Lausanne). 2022;13:1027262. 36589854 10.3389/fendo.2022.1027262 PMC9800885

[28] Sarafidis PA, Ruilope LM. Insulin resistance, hyperinsulinemia, and renal injury: mechanisms and implications. Am J Nephrol. 2006;26(3):232‐244. 16733348 10.1159/000093632

[29] Cohen AJ, McCarthy DM, Stoff JS. Direct hemodynamic effect of insulin in the isolated perfused kidney. Am J Physiol. 1989;257(4\ Pt\ 2):F580‐F585. 2679144 10.1152/ajprenal.1989.257.4.F580

[30] ter Maaten JC, Bakker SJ, Serné EH, ter Wee PM, Donker AJ, Gans RO. Insulin's acute effects on glomerular filtration rate correlate with insulin sensitivity whereas insulin's acute effects on proximal tubular sodium reabsorption correlation with salt sensitivity in normal subjects. Nephrol Dial Transplant. 1999;14(10):2357‐2363. 10528658 10.1093/ndt/14.10.2357

[31] Cusumano AM, Bodkin NL, Hansen BC, et al Glomerular hypertrophy is associated with hyperinsulinemia and precedes overt diabetes in aging rhesus monkeys. Am J Kidney Dis. 2002;40(5):1075‐1085. 12407654 10.1053/ajkd.2002.36348

[32] Weiss O, Anner H, Nephesh I, Alayoff A, Bursztyn M, Raz I. Insulin-like growth factor-I (IGF-I) and IGF-I receptor gene expression in the kidney of the chronically hypoinsulinemic rat and hyperinsulinemic rat. Metabolism. 1995;44(8):982‐986. 7543652 10.1016/0026-0495(95)90093-4

[33] Patinha D, Fasching A, Pinho D, Albino-Teixeira A, Morato M, Palm F. Angiotensin II contributes to glomerular hyperfiltration in diabetic rats independently of adenosine type I receptors. Am J Physiol Renal Physiol. 2013;304(5):F614‐F622. 23283998 10.1152/ajprenal.00285.2012 PMC3602706

[34] Guan Z, VanBeusecum JP, Inscho EW. Endothelin and the renal microcirculation. Semin Nephrol. 2015;35(2):145‐155. 25966346 10.1016/j.semnephrol.2015.02.004 PMC5221560

[35] Sebastian SA, Padda I, Johal G. Cardiovascular-Kidney-Metabolic (CKM) syndrome: a state-of-the-art review. Curr Probl Cardiol. 2024;49(2):102344. 38103820 10.1016/j.cpcardiol.2023.102344

[36] Butlen D, Vadrot S, Roseau S, Morel F. Insulin receptors along the rat nephron: [125I] insulin binding in microdissected glomeruli and tubules. Pflügers Arch. 1988;412(6):604‐612. 3211711 10.1007/BF00583761

[37] Welsh GI, Hale LJ, Eremina V, et al Insulin signaling to the glomerular podocyte is critical for normal kidney function. Cell Metab. 2010;12(4):329‐340. 20889126 10.1016/j.cmet.2010.08.015 PMC4949331

[38] Naderpoor N, Lyons JG, Mousa A, et al Higher glomerular filtration rate is related to insulin resistance but not to obesity in a predominantly obese non-diabetic cohort. Sci Rep. 2017;7(1):45522. 28368024 10.1038/srep45522 PMC5377310

[39] Magen D, Halloun R, Galderisi A, Caprio S, Weiss R. Relation of glomerular filtration to insulin resistance and related risk factors in obese children. Int J Obes (Lond). 2022;46(2):374‐380. 34725443 10.1038/s41366-021-01001-2

[40] Nerpin E, Risérus U, Ingelsson E, et al Insulin sensitivity measured with euglycemic clamp is independently associated with glomerular filtration rate in a community-based cohort. Diabetes Care. 2008;31(8):1550‐1555. 18509205 10.2337/dc08-0369 PMC2494665

[41] Wang X, Huang C, Liu Y, Han Y, Hu H. Association of estimated glomerular filtration rate and incident pre-diabetes: a secondary 5-year longitudinal cohort study in Chinese people. Front Endocrinol (Lausanne). 2022;13:965545. 36387884 10.3389/fendo.2022.965545 PMC9648615

[42] Tu L, Hu H, Zhou X, et al Association between estimated glomerular filtration rate and reversion to normoglycemia in people with impaired fasting glucose: a 5-year retrospective cohort study. Eur J Med Res. 2024;29(1):140. 38388456 10.1186/s40001-024-01669-y PMC10882936

[43] Guder WG, Ross BD. Enzyme distribution along the nephron. Kidney Int. 1984;26(2):101‐111. 6094907 10.1038/ki.1984.143

[44] Stumvoll M, Meyer C, Perriello G, Kreider M, Welle S, Gerich J. Human kidney and liver gluconeogenesis: evidence for organ substrate selectivity. Am J Physiol. 1998;274(5):E817‐E826. 9612239 10.1152/ajpendo.1998.274.5.E817

[45] Nakamura M, Tsukada H, Seki G, et al Insulin promotes sodium transport but suppresses gluconeogenesis via distinct cellular pathways in human and rat renal proximal tubules. Kidney Int. 2020;97(2):316‐326. 31735358 10.1016/j.kint.2019.08.021

[46] Nakamura N, Matsui T, Ishibashi Y, Yamagishi S. Insulin stimulates SGLT2-mediated tubular glucose absorption via oxidative stress generation. Diabetol Metab Syndr. 2015;7(1):48. 26023321 10.1186/s13098-015-0044-1 PMC4447012

[47] Rahmoune H, Thompson PW, Ward JM, Smith CD, Hong G, Brown J. Glucose transporters in human renal proximal tubular cells isolated from the urine of patients with non-insulin-dependent diabetes. Diabetes. 2005;54(12):3427‐3434. 16306358 10.2337/diabetes.54.12.3427

[48] Sharma R, Kumari M, Prakash P, Gupta S, Tiwari S. Phosphoenolpyruvate carboxykinase in urine exosomes reflect impairment in renal gluconeogenesis in early insulin resistance and diabetes. Am J Physiol Renal Physiol. 2020;318(3):F720‐F731. 32036699 10.1152/ajprenal.00507.2019

[49] Shilpasree AS, Patil VS, Revanasiddappa M, Patil VP, Ireshnavar D. Renal dysfunction in prediabetes: confirmed by glomerular hyperfiltration and albuminuria. J Lab Physicians. 2021;13(3):257‐262. 34602791 10.1055/s-0041-1731107 PMC8478507

[50] Echouffo-Tcheugui JB, Narayan KM, Weisman D, Golden SH, Jaar BG. Association between prediabetes and risk of chronic kidney disease: a systematic review and meta-analysis. Diabetes Med. 2016;33(12):1615‐1624. 10.1111/dme.13113 26997583

[51] Atiquzzaman M, Er L, Djurdjev O, et al Implications of implementing the 2021 CKD-EPI equation without race on managing patients with kidney disease in British Columbia, Canada. Kidney Int Rep. 2024;9(4):830‐842. 38765563 10.1016/j.ekir.2024.01.039 PMC11101769

[52] Jiang Y, Owei I, Wan J, Ebenibo S, Dagogo-Jack S. Adiponectin levels predict prediabetes risk: the Pathobiology of Prediabetes in A Biracial Cohort (POP-ABC) study. BMJ Open Diabetes Res Care. 2016;4(1):e000194. 10.1136/bmjdrc-2016-000194 PMC4800069 27026810

[53] Owei I, Umekwe N, Provo C, Wan J, Dagogo-Jack S. Insulin-sensitive and insulin-resistant obese and non-obese phenotypes: role in prediction of incident pre-diabetes in a longitudinal biracial cohort. BMJ Open Diabetes Res Care. 2017;5(1):e000415. 10.1136/bmjdrc-2017-000415 PMC5574414 28878939

[54] Liu Z, Asuzu P, Patel A, Wan J, Dagogo-Jack S. Association of bone mineral density with prediabetes risk among African-American and European-American adult offspring of parents with type 2 diabetes. Front Endocrinol (Lausanne). 2023;13:1065527. 36686435 10.3389/fendo.2022.1065527 PMC9849381

[55] Everett M, Rushing N, Asuzu P, Wan J, Dagogo-Jack S. Association of urinary albumin-to-creatinine ratio with cardiometabolic risk markers and pre-diabetes in adults with normoglycemia, normoalbuminuria, and normotension with parental type 2 diabetes. BMJ Open Diabetes Res Care. 2024;12(1):e003609. 10.1136/bmjdrc-2023-003609 PMC10806903 38233076

